# Comfrey (*Symphytum* spp.) as a feed supplement in pig nutrition contributes to regional resource cycles

**DOI:** 10.1016/j.scitotenv.2021.148988

**Published:** 2021-11-20

**Authors:** Michael Oster, Henry Reyer, Jonas Keiler, Elizabeth Ball, Christina Mulvenna, Siriluck Ponsuksili, Klaus Wimmers

**Affiliations:** a Leibniz Institute for Farm Animal Biology (FBN), Wilhelm-Stahl-Allee 2, 18196 Dummerstorf, Germany; b Department of Anatomy, Rostock University Medical Center, Gertrudenstrasse 9, 18057 Rostock, Germany; c Agri-Food and Biosciences Institute, Large Park, Hillsborough Co. Down BT26 6DR, UK; d Faculty of Agricultural and Environmental Sciences, University Rostock, Justus-von-Liebig-Weg 6, 18059 Rostock, Germany

**Keywords:** Animal nutrition, Monogastrics, Nutrient cycle, Phosphorus cycle, Plant minerals, Protein plants

## Abstract

In smallholder agriculture, the fast-growing and perennial accumulator plant comfrey (*Symphytum* spp.) was used to supply pigs with protein and minerals. Comfrey leaves show similar values in dry matter as soybean or blue lupine in crude protein content, but much higher levels of calcium and phosphorus. However, in terms of increased efficiency in animal husbandry, comfrey has been displaced by mainly soybean and cereals. Due to its profile of macro- and micronutrients the use of comfrey could have the potential to re-establish local resource cycles and help remediate over-fertilized soils. The aim of the study was to evaluate whether a modern pig breed accepts a continuous feed supplement of dried comfrey leaves. After an initial adaptation period post-weaning, German Landrace piglets were subjected to either a standard control diet or a diet supplemented with 15% dried comfrey leaves for 4 weeks. Body weight was reduced in comfrey-supplemented piglets compared to controls, which might be attributed to reduced palatability in the experimental setting. Nevertheless, comfrey-supplemented piglets exhibited adequate bone mineralization and intestinal integrity. The microbiome profile in feces and digesta revealed higher diversity in comfrey-supplemented piglets compared to controls, with pronounced effects on the abundances of *Treponema* and *Prevotella*. This may be due to described bio-positive components of the comfrey plant, as data suggest that the use of comfrey leaves may promote intestinal health. Digestive tract phosphorus levels were reduced in piglets receiving comfrey supplementation, which may ultimately affect phosphorus levels in manure. Results indicate that comfrey leaves could serve as a feed component in integrated agricultural systems to establish regional nutrient cycles. The trial provides a basis for further work on comfrey as a regionally grown protein source and effective replacement for rock mineral supplements.

## Highlights

• Comfrey can be used in mixed crop-livestock farming systems.
• Potential for local resource cycling emphasizing phosphorus and protein
• Comfrey as a feed crop links sustainable soil management and pig farming
• Contribution to remediate currently over-fertilized soils
• Comfrey leaves showed beneficial effects on intestinal health in pigs.

## Graphical abstract

**Figure:**
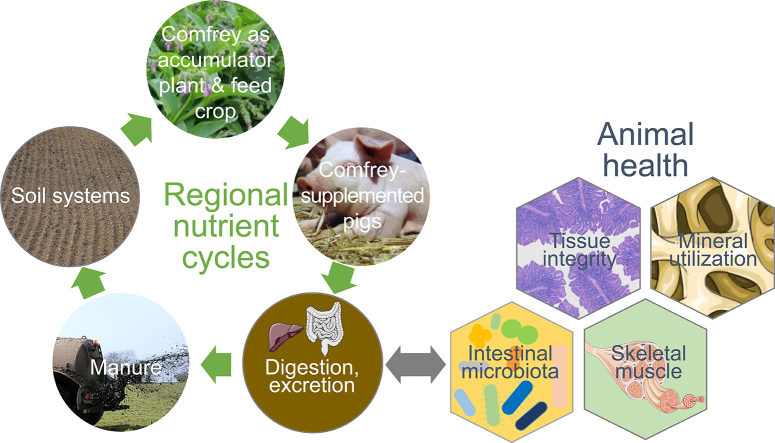

## 1 Introduction

Finite resources and environmental burdens associated with animal emissions demand the prevention of irreversible losses [Campbell et al., 2017]. In view of the current global issues regarding the world food system and thereby fragmented nutrient cycles as well as the accumulation of important minerals such as phosphorus in intensively used agricultural soil, interest in regional nutrient cycles and alternative feed sources have revived [Janes-Bassett et al., 2020; Jarvie et al., 2015; Collette et al., 2011].

In this context, the accumulator plant comfrey (*Symphytum* spp.) has been recognized for its ability to provide minerals in a bioavailable form [Robinson, 1983]. Comfrey varieties are classified in the *Boraginaceae* family and are known as high-yielding, perennial, drought-resistant plants with a height of up to 1.5 m and an extensive root system that develops about 1 m deep into the soil [Robinson, 1983]. The comfrey root is widely recognized for its therapeutic value, especially for improving wound healing [Dähnhardt et al., 2020], osteoarthritis [Grube et al., 2007; Smith and Jacobson, 2011], and its anti-inflammatory properties [Seigner et al., 2019; Trifan et al., 2020a]. The analyses of the aerial parts of comfrey also showed interesting amounts of ingredients for the nutrition of farm animals [Robinson, 1983]. Specifically, comfrey leaves showed 186 g/kg crude ash, 352 g/kg crude protein (285 g/kg digestible protein), 27 g/kg crude fat, 126 g/kg crude fiber, 10.8 g/kg calcium (Ca), 6.9 g/kg phosphorus (P), and 64.9 g/kg potassium (K) in dry matter [Oster et al., 2020], a profile which is consistent with reports from varying climates [Robinson, 1983; Bareeba et al., 1992]. Consequently, comfrey leaves have already been successfully supplemented to diets of broiler chickens as a regional produced protein and mineral source [Oster et al., 2020]. However, knowledge of the use of comfrey in animal nutrition was recognized much earlier, as evidenced by its promotion as a field crop to provide sustenance for pigs during the wartime winters of World War I [Lupus, 1917]. The occasional use of comfrey as farm animal feed has persisted until today [Vogl et al., 2016]. Indeed, traditional forage plants such as comfrey could be revisited, with their application and impact supported by molecular data.

There is an increasing body of evidence about the nutrient and phytochemical profile of comfrey varieties, which suggested beneficial pharmacological effects [Staiger, 2012; Trifan et al., 2020b]. This includes allantoin for tissue regeneration [Savić et al., 2015; Lee et al., 2010], phenolics exhibiting anti-oxidative properties [Nastić et al., 2020; Sarikurkcu et al., 2019], and compounds that exhibit inhibitory effects on gastrointestinal enzymes [Varvouni et al., 2020; Zengin et al., 2021]. Its bioactive compounds may affect the digestive capacity and the development of intestinal microbiota in monogastrics. Indeed, it is known from peasant tradition that comfrey is effective to treat diarrhea in piglets. However, comfrey is also known to contain anti-nutritive constituents such as pyrrolizidine alkaloids, which are found mainly in the root but also in the aerial parts [Awang et al., 1993; Stegemann et al., 2019]. Migration of pyrrolizidine alkaloids through human skin has been shown to be very low [Kuchta and Schmidt, 2020]. When administered in high quantities, the pyrrolizidine alkaloids extracted from comfrey root have been shown to be liver-toxic in poultry [Brown et al., 2016]. However, a detailed review of case reports from recent decades shows that harm due to moderate comfrey consumption has been rarely demonstrated in human [Avila et al., 2020]. Interestingly, breeding comfrey for reduced levels of PA or higher levels of allantoin or polyphenols could result in greater safety and efficacy [Ruzicka et al., 2021].

The objective of the present feasibility study was to evaluate whether pigs accept a feed supplement of 15% dried comfrey leaves from regional cultivars. Dietary effects of this regionally grown feed crop are studied via a comprehensive phenotyping of piglets comprising measurements for growth, performance, tissue integrity, meat quality, microbiome composition, and mineral excretion in an array of tissues such as bone, muscle, liver, and the gastrointestinal tract. The hypotheses of this study were that (i) due to the high mineral content, the use of aerial parts of comfrey could contribute to effectively replace calcium phosphate supplements in livestock feed formulation and (ii) the use of comfrey reduces emissions in farm animal husbandry. The cultivation and application of comfrey would thereby serve to remediate over-fertilized soils with phosphorus excess and establish regional resource cycles.

## 2 Materials and methods

### 2.1 Animals, diets, and sample collection

The study was approved by the Animal Welfare Committee of the FBN and by the Ethics Committee of the State of Mecklenburg-Western Pomerania, Germany (LALLF 7221.3-1-052/18) and complies with the ARRIVE guidelines. The experimental design comprised 20 German Landrace piglets which were obtained from 5 litters. At weaning (28 days), two male and two female piglets per litter were randomly selected. Piglets were assigned to one of two groups of each 10 animals balanced for litter and gender. Animals were kept in pens each consisting of 5 animals. All piglets were kept on control diets for an adaptation period of 1 week which met requirements [AfBN, 2006]. From day 35, piglets received either a standard control diet (CTRL diet) or a diet (COM diet) supplemented with 15% dried comfrey leaves with the corresponding proportional reduction of other components as specified in Table A.1.

The dried comfrey leaves were obtained from greenhouse cultivars (OxyGenesis GmbH, Kalkar, Germany). Chemical analyses revealed 186 g/kg crude ash, 352 g/kg crude protein (285 g/kg digestible protein), 27 g/kg crude fat, 126 g/kg crude fiber, 10.8 g/kg calcium (Ca), 6.9 g/kg phosphorus (P), and 64.9 g/kg potassium (K) in dry matter. The plant material was pulverized. Notably, the diet formulation containing the supplementation of dried comfrey leaves was not balanced for metabolizable energy, amino acids, and mineral content as the study was intended as an initial feasibility trial. Diets were offered ad libitum in pelleted form with 4 mm in diameter (Kahl, Reinbek, Germany). Water was supplied ad libitum.

On day 48 and day 61, individual fecal samples were collected between 08 h00 and 09 h00 respectively. Fecal samples were stored at −20 °C until further analyses. On day 62, piglets were stunned by electronarcosis and subsequently slaughtered by exsanguination in the experimental abattoir of the FBN between 07 h30 and 10 h30. Veterinary inspection of the animals before slaughter and of the carcasses and organs after slaughter attested that the animals were without impairment, symptoms of disease or inflammatory and pathological signs. The sampling comprised muscle (*M. longissimus dorsi*), liver, jejunum, and colon content which were immediately collected, snap-frozen in liquid nitrogen, and stored at −80 °C until analyses. Muscle samples originated at the 13th and 14th rib level. The jejunal sections were approximately 4 cm in length and were taken 1.5 m distal from the pyloric junction. Moreover, the left femora were removed and stored at −20 °C until further analyses.

### 2.2 Growth and performance data

Total body weight (BW) was recorded at day 28, day 35, day 42, day 49, day 56, and day 62 (*n* = 20). Additionally, feed intake was recorded weekly on a pen basis to calculate daily feed intake (DFI), average daily weight gain (ADG), and feed conversion ratio (FCR) for the entire experimental period.

### 2.3 Bone characteristics

The stored femora (*n* = 20) were thawed at room temperature. Individual bones were weighed and bone length and maximal diameter at mid-diaphysis were recorded. Bones were subjected to a breaking strength test [Crenshaw et al., 1981] in which the bones were exposed to a specific stress using a 3-point bending jig (Instron 3366, Instron, High Wycombe, Bucks, UK). The load cell applied a weight of 100 kg at a crosshead speed of 25 mm/min. The force of an attached anvil measuring 50 mm in length and 10 mm in width was placed at the midpoint of the identical facial plane of each bone until the bone failed (determined automatically by the Bluehill software, version 3, Instron). Moreover, femora were subjected to dual energy x-ray absorptiometry (DXA) scans using a STRATOS dR (DMS, Gallargues-le-Montueux, France). The region of interest represented both cancellous and cortical bone parts. Raw data were analyzed using the 3D-DX1 and Medix DR software (Medix DR, Medilink, Mauguio, France) to quantify bone mineral content (BMC) and bone mineral density (BMD).

### 2.4 Meat quality traits

Samples of the *longissimus dorsi* muscle (*n* = 20) taken from the 13th and 14th rib were subjected to analysis of the chemical composition. The content of water, lipids, protein and ash was analyzed by standard methods [Helvich, 1990]. For carcass analyses, measurements of the pH were taken at 45 min and 24 h *postmortem* via a probe electrode (pH-Star, Matthaeus, Pöttmes, Germany). Moreover, measurements of the conductivity were recorded at 45 min and 24 h *postmortem* (LFStar, Matthaeus). Muscle colour was determined with an OPTO-Star device after a chilling period of 24 h at 4 °C (Matthaeus). Moreover, meat colors L* (brightness), a* (redness), and b* (yellowness) were analyzed using a CR-300 device (Minolta AG, Langenhagen, Germany). The drip loss was measured as the weight loss of a foil-wrapped meat sample of 50 g following a 24 h rest at 4 °C as described previously [Honikel, 1998]. Impedance (P*y*) was measured 24 h *postmortem* using the Meat Check 150 device (Sigma Electronic GmbH, Erfurt, Germany).

### 2.5 Quantification of pyrrolizidine alkaloids

To account for dietary exposure related to pyrrolizidine alkaloids, the analyses included the quantification of 28 pyrrolizidine alkaloids in dried comfrey leaves and animal tissues via online- solid phase extraction coupled with liquid-chromatography tandem mass spectrometry (SPE-LC-MS/MS; Institute Kirchhoff Berlin GmbH, Berlin, Germany). Pooled samples of livers and muscles of the experimental groups were subjected to an analysis of pyrrolizidine alkaloids at a detection limit at 5 μg/kg (Table A.2).

### 2.6 Intestinal histology in jejunum

**Fig. 1:**
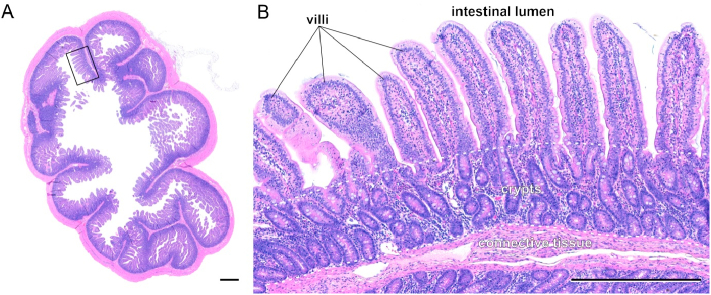
Histological cross section of a representative hematoxylin and eosin (HE)-stained slice of the porcine jejunum (A). Rectangle indicates a representative area along an intestinal circular plica (Kerckring fold) used for analyzing intestinal morphology traits including villus height, villus width and crypt depth (B). Dashed lines indicate the crypt depth. Scale bar = 1 mm (A), 500 μm (B).

The dissected jejunal samples (*n* = 20) were washed in phosphate-buffered saline and subjected to fixation overnight with 3.7% buffered paraformaldehyde. Segments of about 1 cm length were obtained from each sample. Segment processing included a dehydration step using a series of increasing concentrations of ethanol, a transfer into xylol and an embedding in paraffin. Serial sections of 5 μm in thickness were prepared via a microtome (Leica RM2255). The histological sections were deparaffinized and stained with hematoxyline and eosin (HE). Microphotographs were generated with a bright field microscope (Zeiss Axio Imager M2). The microphotographs (*n* = 20) were analyzed with the Fiji/ImageJ software to quantify morphological traits (https://imagej.net/Fiji). Villus height, villus width, and crypt depth of jejunal samples were quantified by ten individual values each (Fig. 1). The jejunal villus to crypt ratio (VCR) was determined [Jeurissen et al., 2002].

### 2.7 16S rRNA profiling

Individual DNA was extracted from feces samples at day 48 (*n* = 20) and day 61 (*n* = 18) as well as from colon digesta sampled at day 62 (n = 20) using the DNeasy PowerLyzer PowerSoil Kit (QIAGEN, Hilden, Germany). In addition to manufactures instructions, the samples were incubated for 10 min at 70 °C and 10 min at 95 °C before bead beating with Precellys 24 homogenizer (PEQLab Biotechnology GmbH, Darmstadt, Germany). The V4 region of the 16S rRNA gene was amplified by PCR using the SupraTherm Tag polymerase (GeneCraft, Lüdinghausen, Germany) and specific primers (515′F and 806R) containing adapters and indices [Kozich et al., 2013; Hugerth et al., 2014]. Briefly, the PCR comprised an initial denaturation step at 95 °C for 2 min, followed by 30 cycles consisting of denaturation at 95 °C for 30 s, annealing at 55 °C for 60 s and at 72 °C for 90 s, and a final extension at 72 °C for 10 min. Amplicons were prepared in duplicates, combined, and subsequently purified and normalized using a SequalPrep normalization plate (Thermo Fisher Scientific, Darmstadt, Germany). Libraries were sequenced on a HiSeq2500 instrument (Illumina, San Diego, CA).

### 2.8 Mineral contents in digesta

The digesta samples of jejunum (*n* = 20) and colon (n = 20) were freeze dried, milled, and digested via microwave treatment. Total phosphorus and calcium levels were quantified by ICP-OES (UEA Consulting Ltd., Norvich, UK).

### 2.9 Data analyses

Weekly recordings of BW were analyzed via an ANOVA including a within subject test to account for repeated measurements. Specifically, the following model was used: Y_ijk_ = μ + d_i_ + t_j_ + (dt)_ij_ + u_k_ + e_ijk_, where Y_ijk_ is the response variable, μ represents the overall mean, d_i_ represents the dietary group, t_j_ represent the time point, u_k_ represents the individual animal, and e_ijk_ represents the residual error. Individual traits for initial BW, bone development, meat quality, intestinal microanatomy, and mineral contents in digesta were analyzed by a linear model (Y_ijk_ = μ + d_i_ + f_j_ + g_k_ + e_ijk_), considering overall mean (μ), dietary group (d_i_), family (f_j_), gender (g_k_), and e_ijk_ as residual error (R language, package lmerTest, v3.1-2). The BW or femoral weight determined on day 62 were used as a covariates where indicated in the result section. Differences were considered significant at *P* ≤ 0.05. For the analysis of the 16S rRNA sequencing, amplicon reads were processed in the mothur software (v1.44.1) and aligned to the Silva reference database (release 138). Operational taxonomic units (OTU) were generated at a sequence identity of 97%. One sample assigned to feces at day 48 was excluded due to low read counts. After subsampling of each sample to 271,091 reads, dietary differences were analyzed at genus level using a Welch's *t*-test within STAMP (v2.1.3) [Parks et al., 2014]. Genera with a Benjamini-Hochberg-adjusted *P*-value <0.05 and a difference between groups in relative abundance above 1% were considered. The significance of convergence between samples assigned to the respective dietary groups was calculated by the analysis of similarities (ANOSIM) approach (R language, package vegan, v2.5–7). Furthermore, the inverse Simpson Index was calculated for feces and digesta samples to account for dietary effects on microbial diversity.

## 3 Results

### 3.1 Growth and performance

**Fig. 2:**
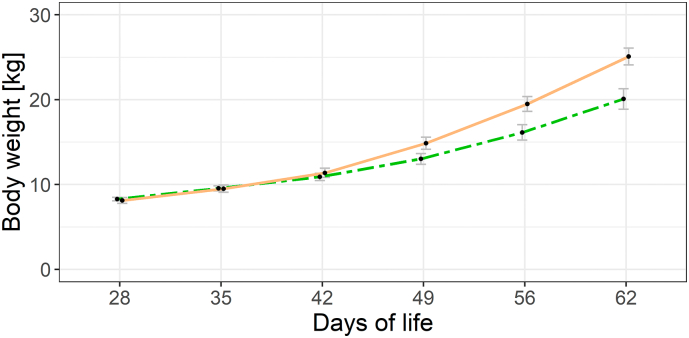
Growth data of piglets fed a standard control diet (CTRL; solid line, orange) and a diet supplemented with 15% dried comfrey leaves (COM; dashed line, green) throughout the experimental period. Data are presented as mean ± SE. (For interpretation of the references to colour in this figure legend, the reader is referred to the web version of this article.)

At day 28, the BW was unaltered between the dietary groups (CTRL: 8.12 ± 0.34 kg; COM: 8.29 ± 0.21 kg; *p* = 0.637). There were no significant differences in BW between groups throughout the post-weaning adaptation period (Fig. 2, Table 1). However, the divergent feeding showed a significantly reduced growth performance in piglets supplemented with dried comfrey leaves from the second week on trial (day 49) until the end of the trial at day 62 (Fig. 2, Table 1). The pen-wise calculations (mean ± SE) showed lowered values for DFI in supplemented piglets compared to the control group (557 ± 3 g vs. 755 ± 3 g), which was reflected in lower ADG (376 ± 2 g vs. 556 ± 4 g) and higher FCR values (1.48 ± 0.01 g/g vs. 1.36 ± 0.01 g/g).

### 3.2 Bone development

No statistically significant differences in femur weight, length and diameter nor in mechanical properties (femur load) and microstructure (bone mineral content, bone mineral density) were observed between the dietary groups (Table 2).

### 3.3 Meat quality traits

The content of water, lipids, protein, and ash in the *longissimus dorsi* muscle was unaltered between pigs supplemented with 15% comfrey leaves compared to control fed animals at day 62 (Table 2). Analyses revealed lowered pH values at 45 min *postmortem* and increased pH values at 24 h *postmortem* in muscle samples of comfrey-supplemented pigs compared to controls. The comfrey supplementation prompted significantly lower values for impedance in muscle samples. Moreover, values for OPTO-Star and a* (redness) were decreased whereas values for L* (lightness) were increased in muscle samples of comfrey-supplemented piglets compared to controls. The measurements for conductivity, b* (yellowness), and drip loss in muscle were unaffected by diet.

### 3.4 Pyrrolizidine alkaloid exposure

The dried comfrey leaves contained a total of 137.0 μg/g DM of pyrrolizidine alkaloids (Table A.2) which corresponds to approximately 20.5 μg/g DM in the comfrey-supplemented diet. Quantified pyrrolizidine alkaloids were below the detection limit in liver and muscle (<5 μg/kg tissue) except for lycopsamine which showed concentrations for muscle and liver of 6 μg/kg and 54 μg/kg, respectively.

### 3.5 Intestinal microanatomy

The intestinal characteristics of jejunal villus height, villus width, crypt depth, and VCR were unaffected by diet (Table 3).

### 3.6 Mineral contents in digesta

Total phosphorus content was significantly reduced in the jejunum digesta of comfrey-supplemented piglets compared with controls, whereas it was not significantly affected in the colon digesta (Table 4). In contrast, total calcium level was unaffected by diet in the jejunum digesta, but was significantly increased in the colon digesta in the comfrey-supplemented piglets compared to the control animals.

### 3.7 Microbiota composition

**Fig. 3:**
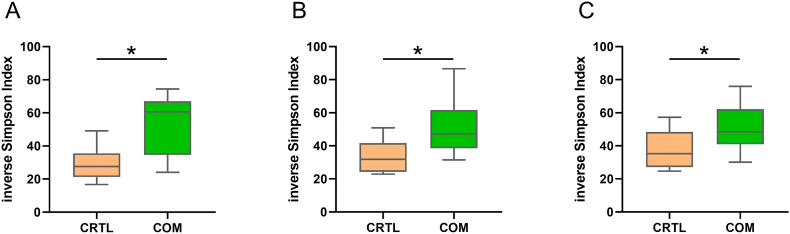
Inverse Simpson Index as a measure of microbial diversity calculated for feces at day 48 (A), feces at day 61 (B), and colon digesta samples at day 62 (C) received from piglets fed a standard control diet (CRTL, orange) and a diet supplemented with 15% dried comfrey leaves (COM, green). *p < 0.05. (For interpretation of the references to colour in this figure legend, the reader is referred to the web version of this article.)

**Fig. 4:**
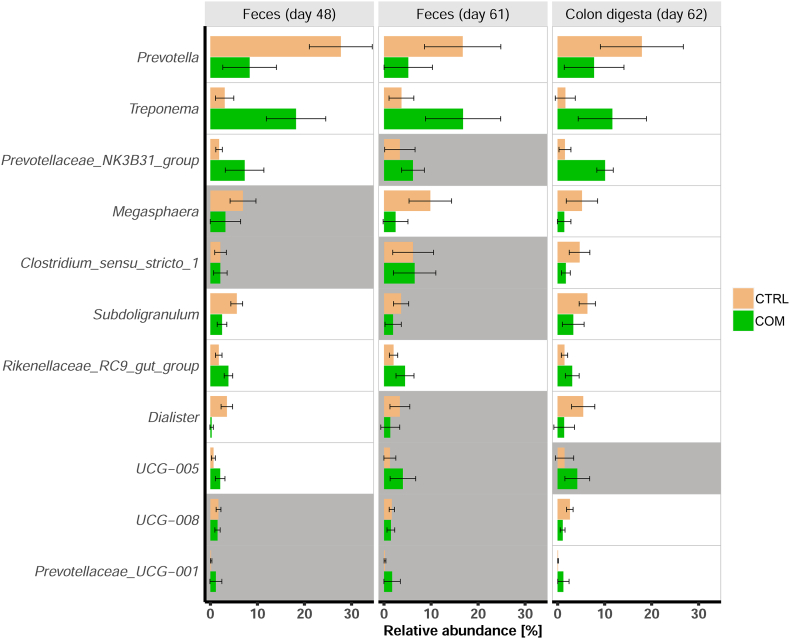
Relative abundances of genera in feces and colon digesta received from piglets fed a standard control diet (CRTL; orange) and a diet supplemented with 15% dried comfrey leaves (COM, green). Genera that differ significantly (adjusted p < 0.05) between dietary groups in a given comparison appear highlighted in white. Data are presented as mean ± SD. (For interpretation of the references to colour in this figure legend, the reader is referred to the web version of this article.)

The analysis of similarities (ANOSIM) resulted in a clear separation of microbial profiles between the dietary groups (feces day 48: *R* = 0.81, *p* < 0.001; feces day 61: *R* = 0.62, *p* < 0.001; colon digesta: *R* = 0.72, *p* < 0.001). In addition, the calculated inverse Simpson Indices showed higher values in the samples from comfrey-supplemented piglets compared to controls, which was effectively the case in all comparisons of feces and colon digesta samples (Fig. 3). Consistently, measurements across specimens and time points also revealed a consistent pattern at the level of genera (Fig. 4, Table A.3). Specifically, *Prevotella* was significantly less abundant in feces and digesta samples of piglets which have received the comfrey supplementation compared with animals fed the control diet. In contrast, *Treponema* and *Rikenellaceae_RC9_gut_group* showed a significantly higher abundance across feces and digesta samples in piglets supplemented with comfrey compared to controls. Moreover, the comfrey supplementation resulted in a higher abundance of *Prevotellaceae_NK3B31_group* but lower abundance of *Subdoligranulum* and *Dialister* in feces (day 48) and colon digesta compared to controls. The genera *Megasphaera*, *Clostridium_sensu_stricto_1*, and *UCG-008* were less abundant, whereas *Prevotellaceae_UCG-001* showed higher abundance in colon digesta of piglets offered the comfrey supplementation. *UCG-005* was higher abundant in feces (day 48) of comfrey-supplemented piglets compared to controls.

**Table A.3:** Relative abundances of genera in feces and the digestive tract. Piglets were fed a standard control diet (CRTL) and a diet supplemented with 15% dried comfrey leaves (COM).

Specimen	Diet	Phylum	Genus	Mean	SD	p-Value	q-Value
Feces (day48)	COM	Firmicutes	Clostridium_sensu_stricto_1	2.11	1.43	0.9561	0.9829
Feces (day48)	CTRL	Firmicutes	Clostridium_sensu_stricto_1	2.14	1.24	0.9561	0.9829
Feces (day48)	COM	Firmicutes	Dialister	0.25	0.37	0.0000	0.0005
Feces (day48)	CTRL	Firmicutes	Dialister	3.48	1.22	0.0000	0.0005
Feces (day48)	COM	Firmicutes	Megasphaera	3.19	3.23	0.0214	0.0562
Feces (day48)	CTRL	Firmicutes	Megasphaera	6.93	2.75	0.0214	0.0562
Feces (day48)	COM	Bacteroidota	Prevotella	8.33	5.72	0.0000	0.0003
Feces (day48)	CTRL	Bacteroidota	Prevotella	27.72	6.68	0.0000	0.0003
Feces (day48)	COM	Bacteroidota	Prevotellaceae_NK3B31_group	7.27	4.11	0.0056	0.0246
Feces (day48)	CTRL	Bacteroidota	Prevotellaceae_NK3B31_group	1.83	0.70	0.0056	0.0246
Feces (day48)	COM	Bacteroidota	Prevotellaceae_UCG-001	1.15	1.27	0.0597	0.1113
Feces (day48)	CTRL	Bacteroidota	Prevotellaceae_UCG-001	0.17	0.14	0.0597	0.1113
Feces (day48)	COM	Bacteroidota	Rikenellaceae_RC9_gut_group	3.83	0.91	0.0001	0.0017
Feces (day48)	CTRL	Bacteroidota	Rikenellaceae_RC9_gut_group	1.76	0.69	0.0001	0.0017
Feces (day48)	COM	Firmicutes	Subdoligranulum	2.47	1.02	0.0000	0.0008
Feces (day48)	CTRL	Firmicutes	Subdoligranulum	5.58	1.25	0.0000	0.0008
Feces (day48)	COM	Spirochaetota	Treponema	18.21	6.33	0.0001	0.0017
Feces (day48)	CTRL	Spirochaetota	Treponema	3.03	1.93	0.0001	0.0017
Feces (day48)	COM	Firmicutes	UCG-005	2.07	1.03	0.0042	0.0209
Feces (day48)	CTRL	Firmicutes	UCG-005	0.63	0.41	0.0042	0.0209
Feces (day48)	COM	Firmicutes	UCG-008	1.50	0.57	0.4186	0.5060
Feces (day48)	CTRL	Firmicutes	UCG-008	1.72	0.51	0.4186	0.5060
Feces (day61)	COM	Firmicutes	Clostridium_sensu_stricto_1	6.50	4.53	0.8889	0.9352
Feces (day61)	CTRL	Firmicutes	Clostridium_sensu_stricto_1	6.18	4.34	0.8889	0.9352
Feces (day61)	COM	Firmicutes	Dialister	1.32	2.00	0.0690	0.1620
Feces (day61)	CTRL	Firmicutes	Dialister	3.37	2.12	0.0690	0.1620
Feces (day61)	COM	Firmicutes	Megasphaera	2.45	2.64	0.0029	0.0367
Feces (day61)	CTRL	Firmicutes	Megasphaera	9.84	4.52	0.0029	0.0367
Feces (day61)	COM	Bacteroidota	Prevotella	5.15	5.13	0.0069	0.0438
Feces (day61)	CTRL	Bacteroidota	Prevotella	16.72	8.11	0.0069	0.0438
Feces (day61)	COM	Bacteroidota	Prevotellaceae_NK3B31_group	6.16	2.42	0.0808	0.1814
Feces (day61)	CTRL	Bacteroidota	Prevotellaceae_NK3B31_group	3.37	3.24	0.0808	0.1814
Feces (day61)	COM	Bacteroidota	Prevotellaceae_UCG-001	1.72	1.75	0.0298	0.1002
Feces (day61)	CTRL	Bacteroidota	Prevotellaceae_UCG-001	0.20	0.19	0.0298	0.1002
Feces (day61)	COM	Bacteroidota	Rikenellaceae_RC9_gut_group	4.46	1.91	0.0045	0.0458
Feces (day61)	CTRL	Bacteroidota	Rikenellaceae_RC9_gut_group	2.02	0.89	0.0045	0.0458
Feces (day61)	COM	Firmicutes	Subdoligranulum	1.93	1.73	0.0623	0.1573
Feces (day61)	CTRL	Firmicutes	Subdoligranulum	3.62	1.62	0.0623	0.1573
Feces (day61)	COM	Spirochaetota	Treponema	16.80	8.00	0.0007	0.0235
Feces (day61)	CTRL	Spirochaetota	Treponema	3.71	2.63	0.0007	0.0235
Feces (day61)	COM	Firmicutes	UCG-005	4.01	2.71	0.0180	0.0792
Feces (day61)	CTRL	Firmicutes	UCG-005	1.25	1.27	0.0180	0.0792
Feces (day61)	COM	Firmicutes	UCG-008	1.46	0.82	0.5986	0.7751
Feces (day61)	CTRL	Firmicutes	UCG-008	1.65	0.55	0.5986	0.7751
Colon (day 62)	COM	Firmicutes	Clostridium_sensu_stricto_1	1.75	0.93	0.0031	0.0156
Colon (day 62)	CTRL	Firmicutes	Clostridium_sensu_stricto_1	4.66	2.19	0.0031	0.0156
Colon (day 62)	COM	Firmicutes	Dialister	1.40	2.20	0.0018	0.0113
Colon (day 62)	CTRL	Firmicutes	Dialister	5.44	2.48	0.0018	0.0113
Colon (day 62)	COM	Firmicutes	Megasphaera	1.40	1.44	0.0086	0.0334
Colon (day 62)	CTRL	Firmicutes	Megasphaera	5.18	3.33	0.0086	0.0334
Colon (day 62)	COM	Bacteroidota	Prevotella	7.75	6.35	0.0125	0.0376
Colon (day 62)	CTRL	Bacteroidota	Prevotella	17.90	8.82	0.0125	0.0376
Colon (day 62)	COM	Bacteroidota	Prevotellaceae_NK3B31_group	10.08	1.76	0.0000	0.0000
Colon (day 62)	CTRL	Bacteroidota	Prevotellaceae_NK3B31_group	1.55	1.27	0.0000	0.0000
Colon (day 62)	COM	Bacteroidota	Prevotellaceae_UCG-001	1.23	1.20	0.0190	0.0499
Colon (day 62)	CTRL	Bacteroidota	Prevotellaceae_UCG-001	0.08	0.07	0.0190	0.0499
Colon (day 62)	COM	Bacteroidota	Rikenellaceae_RC9_gut_group	3.11	1.45	0.0088	0.0319
Colon (day 62)	CTRL	Bacteroidota	Rikenellaceae_RC9_gut_group	1.46	0.69	0.0088	0.0319
Colon (day 62)	COM	Firmicutes	Subdoligranulum	3.33	2.32	0.0066	0.0278
Colon (day 62)	CTRL	Firmicutes	Subdoligranulum	6.31	1.73	0.0066	0.0278
Colon (day 62)	COM	Spirochaetota	Treponema	11.63	7.24	0.0024	0.0131
Colon (day 62)	CTRL	Spirochaetota	Treponema	1.64	2.12	0.0024	0.0131
Colon (day 62)	COM	Firmicutes	UCG-005	4.18	2.65	0.0245	0.0572
Colon (day 62)	CTRL	Firmicutes	UCG-005	1.48	1.93	0.0245	0.0572
Colon (day 62)	COM	Firmicutes	UCG-008	1.07	0.52	0.0001	0.0017
Colon (day 62)	CTRL	Firmicutes	UCG-008	2.60	0.70	0.0001	0.0017

**Table 1:** Summary of effects mediated by diet, time, and their interaction on growth data of piglets fed a standard control diet (CRTL) and a diet supplemented with 15% dried comfrey leaves (COM). Data are presented on a weekly basis and for the entire experimental period.

Variable	*P* value					
	Day 28–35	Day 35–42	Day 42–49	Day 49–56	Day 56–62	Days35–62
Diet	0.819	0.718	0.167	0.029	0.008	0.041
Time	<0.001	<0.001	<0.001	<0.001	<0.001	<0.001
Diet × time	0.564	0.070	0.014	0.002	<0.001	<0.001

**Table 2:** Femur characteristics and meat quality traits of the longissimus dorsi muscle. Piglets were fed a standard control diet (CRTL) and a diet supplemented with 15% dried comfrey leaves (COM). Data are presented as mean ± SE.

Item	Unit	CRTL diet	COM diet	P value
Femur characteristics				
Femur weight^a^	g	121.3 ± 4.1	102.9 ± 5.9	0.314
Femur length^a^	mm	117.1 ± 1.2	109.7 ± 2.0	0.403
Femur diameter^a^	mm	15.1 ± 0.2	14.8 ± 0.3	0.064
Femur max. load^b^	kg	170.9 ± 9.3	147.3 ± 12.2	0.807
Bone mineral content^b^	g	1.47 ± 0.02	1.47 ± 0.02	0.854
Bone mineral density^b^	g/cm^2^	0.75 ± 0.01	0.75 ± 0.01	0.839
Meat quality				
Water content^a^	%	76.66 ± 0.15	77.26 ± 0.19	0.237
Lipid content^a^	%	1.21 ± 0.06	1.37 ± 0.06	0.867
Protein content^a^	%	20.70 ± 0.23	19.82 ± 0.17	0.308
Ash content^a^	%	1.14 ± 0.01	1.16 ± 0.01	0.252
pH (45 min)		6.28 ± 0.06	6.12 ± 0.03	0.013
pH (24 h)		5.43 ± 0.02	5.49 ± 0.02	0.030
Conductivity (45 min)	mS/cm	4.07 ± 0.15	4.04 ± 0.11	0.867
Conductivity (24 h)	mS/cm	2.68 ± 0.09	2.90 ± 0.18	0.149
Impedance (24 h)	P*y*	49.40 ± 2.67	43.70 ± 1.92	0.044
Drip loss	%	3.59 ± 0.28	3.90 ± 0.39	0.450
OPTO-Star		73.17 ± 1.16	70.12 ± 1.27	0.040
L* (lightness)		49.99 ± 0.55	51.58 ± 0.62	0.025
a* (redness)		7.90 ± 0.21	7.22 ± 0.23	0.016
b* (yellowness)		1.26 ± 0.24	1.43 ± 0.18	0.516

a Statistical analyses considered live body weight at day 62.

b Statistical analyses considered femoral weight at day 62.

**Table 3:** Histology traits of jejunum of piglets fed a standard control diet (CRTL) and a diet supplemented with 15% dried comfrey leaves (COM). Data are presented as mean ± SE.

Trait	Unit	CRTL diet	COM diet	P value
Villus height	μm	475.9 ± 24.0	458.3 ± 28.3	0.611
Villus width	μm	153.0 ± 8.5	163.3 ± 9.2	0.345
Crypt depth	μm	292.7 ± 9.3	299.9 ± 21.1	0.735
VCR		1.64 ± 0.09	1.56 ± 0.09	0.510

**Table 4:** Total phosphorus and calcium levels in digesta of piglets fed a standard control diet (CRTL) and a diet supplemented with 15% dried comfrey leaves (COM). Data are presented as mean ± SE.

Trait	Unit	CRTL diet	COM diet	**P value**
Total phosphorus (jejunum)	mg/g	8.39 ± 1.15	5.56 ± 0.79	0.038
Total phosphorus (colon)	mg/g	15.61 ± 0.94	14.01 ± 0.74	0.081
Total calcium (jejunum)	mg/g	6.56 ± 1.29	6.48 ± 1.27	0.925
Total calcium (colon)	mg/g	20.10 ± 0.69	25.63 ± 1.11	<0.001

## 4 Discussion

Balancing environmental conservation, consumer demands and economic constraints is a major challenge, especially in modern animal husbandry. Improved implementation of regional nutrient cycles offers key benefits along the agricultural value chain by enabling finite and valuable resources to be recycled [Willett et al., 2019]. If left unmanaged, pollution easily grows into a supra-regional issue [Cui et al., 2021]. In fact, the use of comfrey as feed crop could be a link between sustainable soil management and regional livestock farming that is demanded by consumers and society. Since comfrey varieties are known to be accumulator plants, its use in soil management strategies is discussed [Du et al., 2019] which might tackle the critical issue of reducing the environmental impact of livestock farming in terms of phosphorus emissions. Indeed, losses of minerals such as phosphorus remain one of the most significant challenges in farm animal husbandry, which has a social dimension in addition to environmental consequences [Campbell et al., 2017; El Wali et al., 2021]. By employing comfrey, there is potential to sustainably integrate currently over-fertilized soils, where large quantities of animal manure are persistently applied, into regional agricultural cycles, while at the same time supplementing other protein-rich crops.

In the current study, the comfrey supplementation was moderately accepted by the pigs. Growth and performance data such as daily feed intake and daily weight gain were lowered compared to the control group. However, as the trial was designed as a feasibility study, no balancing of metabolizable energy, amino acids, and mineral content between experimental diets was performed and no flavor enhancers were used. In addition to the elevated total dietary fiber content (0.42 g/kg DM vs. 0.32 g/kg DM) and the lower energy content of the COM diet (13.2 MJ/kg DM vs. 13.9 MJ/kg DM), reductions in growth parameters are probably driven by the lowered feed intake, which might be attributed to reduced palatability of the diet in the applied experimental setting. Thus, palatability must be one of the primary considerations in upcoming feeding trials with comfrey. It is well documented that eliciting taste stimuli through sweet and umami flavors using molasses, sucrose, or yeast extracts can increase feed intake with positive effects on growth performance [Figueroa et al., 2019; Roura et al., 2008]. In this context, the age of animals should be considered since also chickens subjected to an initial trial with supplemented comfrey leaves in their feed showed reduced performance during the early development, i.e. the first week of life, but could catch up during the grower and finisher phases [Oster et al., 2020]. Studies in pigs showed absent or moderate weight reductions following comfrey supplementation of up to 25% at the finisher stage [Bee et al., 1999]. The obtained results in this study serve as a basis for further trials on supplementing comfrey leaves to pigs. Further work should determine both optima and upper limits of comfrey supplementation during growing and fattening periods in pig husbandry.

Regarding traits on tissue integrity and health, the bone characteristics were unaffected by diet which indicate a maintained mineral homeostasis in comfrey-supplemented pigs (Table 2). From an animal health perspective, this was also evident in bone stability measurements including breaking strength, bone mineral content, and bone mineral density. There was also no dietary effect for most meat quality parameters such as protein content, ash content, conductivity or drip loss. However, measurements of pH, impedance, and meat colour showed differences between dietary groups but ensure a high quality of meat from both groups (Table 2). Nevertheless, results indicate that plant components of comfrey leaves impact on muscle metabolism, which might be attributed to anti-oxidative properties [Nastić et al., 2020; Sarikurkcu et al., 2019]. In addition, analyses of the fatty acid profile in muscle revealed decreased levels of saturated fatty acids (SFA) and increased levels of monounsaturated fatty acids (MUFA) in pigs fed comfrey diets [Bee et al., 1999]. In fact, recently published research showed a wide range of constituents of comfrey plant material, such as allantoin or polysaccharides, whose in-vivo effects require further investigation [Kimel et al., 2020; Shang et al., 2020].

Histological analyses of the jejunum showed no conspicuous lesions with respect to intestinal microanatomical parameters in comfrey-supplemented pigs compared to control fed animals (Table 3). Measurements on villi and crypts were in agreement with results of previous studies performed in pigs [Le Bon et al., 2010]. Results suggest that the daily exposure to the bioactive compounds of comfrey leaves did not result in intestinal atrophy or malabsorption. It is assumed that the tissue integrity and resorption capacity of the jejunum was maintained during the experimental period in all individuals. However, piglets offered the comfrey supplementation showed considerable differences in the microbial composition compared to controls. In fact, the calculated *R* values and inverse Simpson Index in feces and digesta samples account for a higher microbial diversity in comfrey-supplemented piglets compared to control animals (Fig. 3). There is a body of evidence, that higher diversity at the absence of pathogens is associated with beneficial effects and might have, for example, a preventive effect against diarrhea [Rouhani et al., 2020]. This could explain the efficacy attributed to comfrey leaves in treating piglet diarrhea. Specifically, at the phylum level a shift from *Firmicutes* (e.g., *Megasphaera*, *Dialister*, *Subdoligranulum*) in favor of *Spirochaetota* (*Treponema*) appeared in feces and colon digesta of comfrey-fed animals (Fig. 4). Since high abundances of *Firmicutes* have been associated with obesity, their decrease in comfrey-supplemented piglets might be considered benficial for metabolic health [Turnbaugh et al., 2006]. Moreover, the relative abundance of *Prevotella* which belongs to the phylum *Bacteriodota* significantly decreased after comfrey supplementation. It is conceivable that the observed microbial shifts result from different amounts of dietary carbohydrates, minerals such as calcium and pottassium, sulfur, or bioactive components present in comfrey plant material. In fact, comfrey leaves show a low protein to fiber ratio and its proteins exhibit relatively high levels of sulfur-containing amino acids such as cysteine and methionine [Robinson, 1983]. In addition, previous studies reported the phytochemical profile of comfrey to include a number of phenolic compounds [Nastić et al., 2020], which may be involved in remodeling the intestinal microbiota via their conversion to bioactive and bioavailable metabolites [Selma et al., 2009; Lakes et al., 2020]. Interestingly, polyphenols have been shown to affect the growth of species classified as *Firmicutes* and *Bacteroidetes* [Ozdal et al., 2016]. Moreover, polysaccharides have been detected in comfrey [Shang et al., 2020], which serve as fermentation substrates and could also impact on the composition of the microbiota [Xu et al., 2013].

The phosphorus and calcium levels in digesta of jejunum and colon (Table 4) accounts for the divergent nutrient composition (Table A.1). Besides yet unknown effects on mineral digestibility, differences reflect also effects of the reduced feed intake in comfrey-supplemented piglets compared to controls. Indeed, the diet supplemented with comfrey leaves is higher (+13.5%) in calcium (Table A.1), which is finally also reflected in the increased calcium level in the colon digesta (+27.5%; *p* < 0.001) of comfrey-supplemented animals (Table 4). Furthermore, the phosphorus content of the comfrey-supplemented diet is slightly reduced (−3.6%). However, the remarkable reduction of phosphorus content in both jejunum digesta (−33.7%; *p* = 0.038) and colon digesta (−10.2%; *p* = 0.081) might indicate high bioavailability and efficacy strategies in mineral absorption, while lowering phosphorus excretion (Table 4).

The amount of pyrrolizidine alkaloids (Table A.2) corresponds to the range detected in the aerial parts of comfrey in previous reports [Couet et al., 1996]. After a daily exposure of 4 weeks, comfrey-supplemented piglets showed traces of pyrrolizidine alkaloids of 54 μg/kg DM and 6 μg/kg DM in liver and muscle samples, respectively. In view of the fact that the animals subsequently ingested approximately 11.4 mg/d of pyrrolizidine alkaloids during the divergent feeding period depending on a DFI of approximately 557 g, this suggests sophisticated detoxification mechanisms in the growing pig. However, pyrrolizidine alkaloids can be metabolized via the cytochrome P450 family by biotransformation to reactive pyrroles with pronounced toxicity [Stickel and Seitz, 2000]. Although pigs are thought to be vulnerable to pyrrolizidine alkaloids [Rode, 2002], none of the animals in this study showed evidence of intoxication. The current limits for pyrrolizidine alkaloid intake are based only on recommendations of the direct exposure of human subjects [BfR*,* 2013]. However, the results suggest that there is a need to quantify safety margins in animal nutrition and specific animal-based products. This particularly concerns organic farming and pyrrolizidine alkaloid-containing plants in free-range systems [Orantes-Bermejo et al., 2013].

## 5 Conclusions

In summary, comfrey leaves can be considered as feed crop for pigs, as the analyses of traits in bone, jejunum, digesta, feces, liver and muscle indicated maintenance of tissue function and integrity in all animals. Piglets fed a standard diet supplemented with 15% dried comfrey leaves did not show clinical aberrations despite the relatively high exposure of pyrrolizidine alkaloids. The comfrey supplementation promoted considerable shifts in the microbiota composition which revealed to exhibit a high degree of diversity. To overcome the reductions in feed intake, further studies should monitor pig performance traits with flavor enhancer and balanced metabolizable energy levels. The possibilities of using the aerial parts of comfrey in farm animal nutrition should be carefully evaluated as a source of local protein and to effectively replace calcium phosphate supplements as a contribution to achieving sustainable pig farming and establishing regional nutrient cycles in agriculture.

## Funding

This work has received funding from the European Research Area Network (ERA-NET) co-funds on Sustainable Animal Production (SusAn) as part of the PEGaSus project (2817ERA02D). The Leibniz Institute for Farm Animal Biology (FBN) provided own matched funding.

## CRediT authorship contribution statement

**Michael Oster:** Conceptualization, Formal analysis, Investigation, Methodology, Project administration, Visualization, Writing – original draft. **Henry Reyer:** Conceptualization, Data curation, Formal analysis, Investigation, Methodology, Visualization, Writing – review & editing. **Jonas Keiler:** Methodology, Investigation, Visualization, Resources, Writing – review & editing. **Elizabeth Ball:** Methodology, Resources, Writing – review & editing. **Christina Mulvenna:** Methodology, Resources, Writing – review & editing. **Siriluck Ponsuksili:** Methodology, Resources, Data curation, Writing – review & editing. **Klaus Wimmers:** Conceptualization, Methodology, Resources, Writing – review & editing, Supervision, Project administration, Funding acquisition.

## Declaration of competing interest

The authors declare no conflict of interest. The funders had no role in the design of the study; in the collection, analyses, or interpretation of data; in the writing of the manuscript, or in the decision to publish the results.

## Figures and Tables

**Table A.1:** Calculated ingredients and analyzed nutrient composition of the experimental diets during adaptation and grower periods. CRTL – standard control diet; COM – diet supplemented with 15% dried comfrey leaves.

Item	Unit	CRTL diet	COM diet
Calculated			
Barley	%	31.51	26.78
Soybean meal	%	15.74	13.38
Wheat	%	15.55	13.22
Comfrey leaves (94% DM)	%	–	15.00
Concentrate 1232^a^	%	9.50	8.08
Maize	%	9.00	7.65
GreenGoldMix (barley, wheat, maize)	%	7.30	6.21
Powdered milk	%	2.00	1.70
Premix	%	2.00	1.70
Soy protein (Provimi Provisoy)	%	1.50	1.28
Soybean oil	%	1.11	0.94
Beet pulp	%	1.00	0.85
Calcium carbonate (Vitacarb 15)	%	0.80	0.68
MCP - Monocalciumphosphate	%	0.79	0.67
Formic acid/Lactic acid	%	0.50	0.43
Lysine sulfate	%	0.32	0.27
Methionine	%	0.31	0.26
Citric acid	%	0.30	0.26
Threonine	%	0.28	0.23
Fish oil	%	0.20	0.17
Benzoic acid	%	0.20	0.17
Sodium chloride	%	0.10	0.09

Analyzed			
DM	g/kg FM	907	907
Crude ash	g/kg DM	49	67
Crude protein	g/kg DM	177	181
Crude fat	g/kg DM	49	44
Crude fiber	g/kg DM	32	42
Starch	g/kg DM	398	336
Calcium (Ca)	g/kg DM	7.7	8.9
Phosphorus (P)	g/kg DM	5.5	5.3
Sodium (Na)	g/kg DM	2.1	1.3
Magnesium (Mg)	g/kg DM	1.6	1.7
Potassium (K)	g/kg DM	8.3	18.2
ME (pig)	MJ/kg DM	13.9	13.2

FM – fresh matter; DM – dry matter; ME – metabolizable energy.

a Soy protein concentrate, oat flakes, lactose, saccharose, wheat, shea oil, soybeans, palm kernel oil, calcium carbonate.

**Table A.2:** Quantified pyrrolizidine alkaloids in dried comfrey leaves, and pig liver and muscle tissues.

Pyrrolizidin alkaloid	Unit	Concentration in dried comfrey leaves	Concentration in liver (piglets on CRTL diet)	Concentration in muscle (piglets on CTRL diet)	Concentration in liver (piglets on COM diet)	Concentration in muscle (piglets on COM diet)
7-O-Acetylintermedine	μg/kg DM	55	<5	<5	<5	<5
7-O-Acetyllycopsamine	μg/kg DM	1010	<5	<5	<5	<5
7-O-Acetylintermedine N-oxide	μg/kg DM	72	<5	<5	<5	<5
7-O-Acetyllycopsamine N-oxide	μg/kg DM	1740	<5	<5	<5	<5
Echimidine	μg/kg DM	2600	<5	<5	<5	<5
Echimidine N-oxide	μg/kg DM	9500	<5	<5	<5	<5
Erucifoline	μg/kg DM	<5	<5	<5	<5	<5
Erucifoline N-oxide	μg/kg DM	<5	<5	<5	<5	<5
Europine	μg/kg DM	<5	<5	<5	<5	<5
Europine N-oxide	μg/kg DM	<5	<5	<5	<5	<5
Heliotrin	μg/kg DM	<5	<5	<5	<5	<5
Heliotrin N-oxide	μg/kg DM	<5	<5	<5	<5	<5
Intermedine	μg/kg DM	290	<5	<5	<5	<5
Intermedine N-oxide	μg/kg DM	1700	<5	<5	<5	<5
Jacobine	μg/kg DM	<5	<5	<5	<5	<5
Jacobine N-oxide	μg/kg DM	<5	<5	<5	<5	<5
Lasiocarpine	μg/kg DM	<5	<5	<5	<5	<5
Lasiocarpine N-oxide	μg/kg DM	<5	<5	<5	<5	<5
Lycopsamine	μg/kg DM	21,000	<5	<5	54	6
Lycopsamine N-oxide	μg/kg DM	99,000	<5	<5	<5	<5
Monocrotaline	μg/kg DM	<5	<5	<5	<5	<5
Monocrotaline N-oxide	μg/kg DM	<5	<5	<5	<5	<5
Retrorsine	μg/kg DM	<5	<5	<5	<5	<5
Retrorsine N-oxide	μg/kg DM	<5	<5	<5	<5	<5
Senecionine	μg/kg DM	<5	<5	<5	<5	<5
Senecionine N-oxide	μg/kg DM	<5	<5	<5	<5	<5
Seneciphylline	μg/kg DM	<5	<5	<5	<5	<5
Seneciphylline N-oxide	μg/kg DM	<5	<5	<5	<5	<5
Senecivernine	μg/kg DM	<5	<5	<5	<5	<5
Senecivernine N-oxide	μg/kg DM	<5	<5	<5	<5	<5
Senkirkine	μg/kg DM	<5	<5	<5	<5	<5
Trichodesmine	μg/kg DM	<5	<5	<5	<5	<5
